# A new ancient lineage of frog (Anura: Nyctibatrachidae: Astrobatrachinae subfam. nov.) endemic to the Western Ghats of Peninsular India

**DOI:** 10.7717/peerj.6457

**Published:** 2019-03-12

**Authors:** Seenapuram Palaniswamy Vijayakumar, Robert Alexander Pyron, K. P. Dinesh, Varun R. Torsekar, Achyuthan N. Srikanthan, Priyanka Swamy, Edward L. Stanley, David C. Blackburn, Kartik Shanker

**Affiliations:** 1 Centre for Ecological Sciences, Indian Institute of Science, Bangalore, Karnataka, India; 2 Department of Biological Sciences, The George Washington University, Washington, DC, USA; 3 Western Regional Centre, Zoological Survey of India, Pune, Maharashtra, India; 4 Florida Museum of Natural History, University of Florida, Gainesville, FL, USA

**Keywords:** Molecular phylogenetics, *Astrobatrachus kurichiyana* gen et. sp. nov., Natatanura, Microendemism, Astrobatrachinae subfam. nov., Osteology

## Abstract

The Western Ghats (WG) is an escarpment on the west coast of Peninsular India, housing one of the richest assemblages of frogs in the world, with three endemic families. Here, we report the discovery of a new ancient lineage from a high-elevation massif in the Wayanad Plateau of the southern WG. Phylogenetic analysis reveals that the lineage belongs to Natatanura and clusters with Nyctibatrachidae, a family endemic to the WG/Sri Lanka biodiversity hotspot. Based on geographic distribution, unique morphological traits, deep genetic divergence, and phylogenetic position that distinguishes the lineage from the two nyctibatrachid subfamilies Nyctibatrachinae Blommers-Schlösser, 1993 and Lankanectinae Dubois & Ohler, 2001, we erect a new subfamily Astrobatrachinae **subfam. nov.** (endemic to the WG, Peninsular India), and describe a new genus *Astrobatrachus* **gen. nov.** and species, *Astrobatrachus kurichiyana* **sp. nov.** The discovery of this species adds to the list of deeply divergent and monotypic or depauperate lineages with narrow geographic ranges in the southern massifs of the WG. The southern regions of the WG have long been considered geographic and climatic refugia, and this new relict lineage underscores their evolutionary significance. The small range of this species exclusively outside protected areas highlights the significance of reserve forest tracts in the WG in housing evolutionary novelty. This reinforces the need for intensive sampling to uncover new lineages and advance our understanding of the historical biogeography of this ancient landmass.

## Introduction

Ancient lineages hold significant information for understanding the biogeographic past of different regions (Fjeldsaå & Lovett, 1997). A large concentration of old lineages may signify historical refugia that are of great evolutionary and conservation significance. In South Asia, the Western Ghats (WG) escarpment of Peninsular India and the island of Sri Lanka have long been recognized as rainforest refuges (Wallace, 1876; Mani, 1974). These regions are an evolutionary repository for amphibians with hundreds of frog species from nine families: Bufonidae Gray, 1825; Dicroglossidae Anderson, 1871; Micrixalidae Dubois, Ohler & Biju, 2001; Microhylidae Günther, 1858 (1843); Nasikabatrachidae Biju & Bossuyt, 2003; Nyctibatrachidae Blommers-Schlösser, 1993; Ranidae Batsch, 1796; Ranixalidae Dubois, 1987; and Rhacophoridae Hoffman, 1932 (1858) (see Dutta et al., 2004; Van Bocxlaer et al., 2009; Biju et al., 2011, 2014; Vijayakumar et al., 2014; Dinesh et al., 2015; Dahanukar et al., 2016). Three of these frog families (Micrixalidae, Ranixalidae, and Nasikabatrachidae) are endemic to the WG, while a fourth, Nyctibatrachidae, consists of two subfamilies: Nyctibatrachinae which is endemic to the WG, and Lankanectinae which is endemic to Sri Lanka.

This evolutionarily distinct and diverse assemblage of frogs has only been recently uncovered by studies highlighting the radiation of ancient lineages (Roelants, Jiang & Bossuyt, 2004) and high clade-level endemism in the WG and Sri Lanka (Meegaskumbura et al., 2002; Bossuyt et al., 2004). These studies have provided new insights and perspectives, triggering renewed interest within the research community to document and understand frog diversity in the WG. Accordingly, a number of higher-level taxa (e.g., families) have been recognized from the WG, including representatives from Ranixalidae, Nyctibatrachidae, and Micrixalidae, which historically were recognized as subfamilies of Ranidae (Blommers-Schlösser, 1993; Dubois, 2005) but were subsequently elevated to families (Frost et al., 2006). These taxa are part of the larger, primarily Old World natatanuran radiation that diversified across Afrotropical, Madagascan, Palearctic, and Oriental realms (Bossuyt et al., 2006; Wiens et al., 2009; Feng et al., 2017; Yuan et al., 2018).

Here, we report the discovery of a hitherto unknown taxon; an ancient, monotypic, microendemic lineage forming a strongly supported clade with Nyctibatrachidae Blommers-Schlösser, 1993. Based on its geographic distribution, age, phylogenetic position, deep morphological divergence, and distinct habits, we assign the taxon to a new subfamily Astrobatrachinae **subfam. nov.** and genus *Astrobatrachus* **gen. nov.**, and describe a single new species *Astrobatrachus kurichiyana* **sp. nov.** The discovery adds to the list of ancient and monotypic lineages with narrow geographic ranges in the southern massifs of the WG.

## Materials and Methods

### Specimen collection

**Figure 1:**
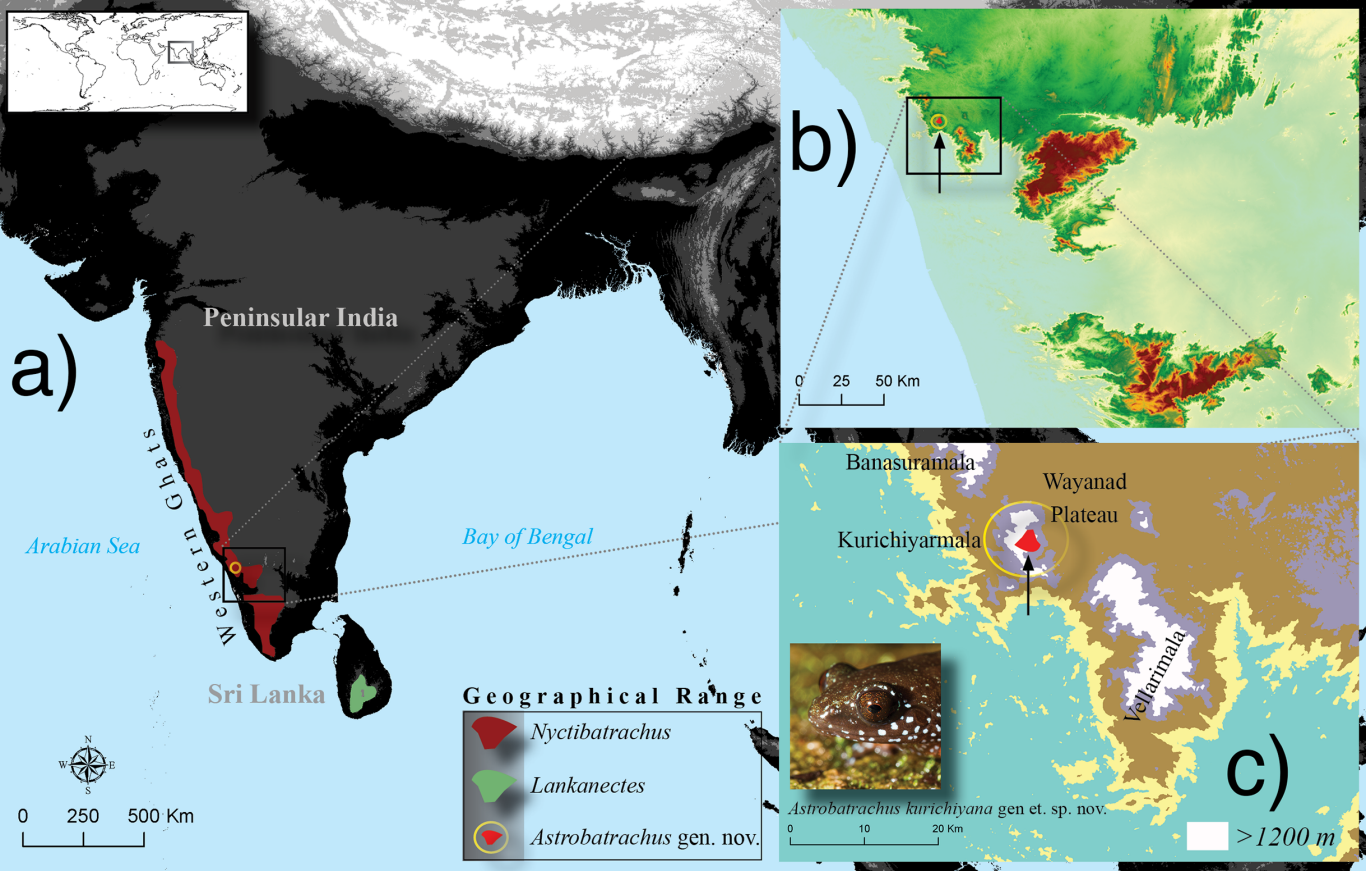
Geographical range (A) of the three genera, *Nyctibatrachus* (Nyctibatrachinae), *Lankanectes* (Lankanectinae) and the new genus *Astrobatrachus* (Astrobatrachinae subfam. nov.). Inset maps show the type locality (B) and the narrow range (C) of *Astrobatrachus kurichiyana* gen et. sp. nov. Photo Credit: S. P. Vijayakumar.

The taxon was discovered during an expedition to an isolated hill range (Kurichiyarmala) located on the western edge of Wayanad Plateau in the south-central region of the WG Escarpment (Fig. 1). The expedition was part of a large-scale survey for amphibians and reptiles across all the major massifs and plateaus in the WG (see Vijayakumar et al., 2014). Individuals were captured in June 2010 during nocturnal searches on the forest floor and in grasslands adjoining forest. One of us (KPD) revisited the site in September 2017 and carried out intensive night sampling to understand the general habits and habitat of the species. A subset of the individuals collected were fixed and stored in 70% alcohol, while others were fixed in 4% formaldehyde and stored in 70% alcohol. Liver tissues were extracted and stored in 95% alcohol before fixing in formaldehyde. Photographs of live individuals, on which color descriptions are based, were taken before euthanizing the individuals. The type specimens have been deposited at the Zoological Survey of India (ZSI), Western Regional Centre (WRC), Pune. Specimens were collected under permit numbers WL12-6574/2006 from the Kerala Forest Department. At the time of the initial study, IISc did not maintain a formal ethics committee for field research conducted under valid permits. All field research was conducted in ethical compliance with the SSAR Guidelines (Beaupre et al., 2004).

### DNA sequencing and alignment

We generated new DNA-sequence data from four specimens using existing primers. We sequenced 16S and RHOD (CESF 1566) and CYTB and RHOD (CESF 1567) (primers from Frost et al., 2006). We also developed new primers for the RAG1 locus: RAG1_Forward_Primer 5′-GATGGAATGGGAGATGTYAGYGAGAA-3′, RAG1_Reverse_Primer 5′-AAAAGTTYTGCRAARCGTTGAGAGTG-3′. These primers amplify and sequence an approximately ∼850 bp piece of the RAG1 locus that is homologous with the fragment sequenced for hundreds of amphibian species in many existing datasets (e.g., Roelants et al., 2007). We also developed new primers for the CXCR4 locus: CXCR4_Ranoidea_Forward 5′-TGGTCTGTGGATGCYGCCATYGGTTGGTA-3′, CXCR4_Ranoidea_Reverse 5′-ATGTAGTAYGGVAGCCARCAGGC-3′. We generated 835 bp of sequence from a third specimen (CESF 2895) for RAG1 and 459 bp for CXCR4.

We used an existing alignment (Jetz & Pyron, 2018—JP hereafter) to place these new taxa in the Amphibian Tree of Life. The JP matrix contains 15 genes (5 mitochondrial, 10 nuclear), including 16S, CYTB, CXCR4, RAG1, and RHOD. The other 10 alignments (12S, BDNF, H3A, NCX1, ND1, ND2, POMC, SIA, SLC8A3, and TYR) were used without modification. The GenBank accession numbers for the existing sequences are given by Jetz & Pyron (2018). Our new sequences have been deposited under the GenBank numbers MK144662, MK153264–MK153268.

The new data were added to the JP alignments using the ‘Profile Align’ feature in Geneious 11.0.4 (Biomatters Ltd., Auckland, NZ). For CYTB and RHOD, there was no length variation, and thus the profiled alignments required no further refinement. For 16S, we realigned the regions overlapping with the new sequences using the MAFFT algorithm (Katoh et al., 2002) in Geneious, under the default settings. Similarly, as CXCR4 and RAG1 contain several indels, we realigned those sequences using the ‘Translation Align’ option with the MAFFT algorithm.

The JP matrix had 4,062 terminals and 15,091 bp. Our final matrix had 4,063 terminals and 15,359 bp. As the samples of the new species did not overlap sufficiently in terms of sampled loci, we concatenated the 16S, CYTB, CXCR4, RAG1, and RHOD data from the three specimens, and included these data as a single composite terminal, for a total of 2,125 bp from five genes. This is justified as all three individuals were sampled from the same site and shared the unique morphological characteristics described below, and overlapping sequences were invariant.

We used PartitionFinder 2 (Lanfear et al., 2017) to estimate the best partitioning strategy, given two ribosomal genes and 13 protein-coding genes for a total of 41 possible partitions including codon positions. We used the--raxml (Stamatakis, 2006) and search = ‘rcluster’ (Lanfear et al., 2014) flags to decrease computational intensity, given the size of the matrix. We restricted the model set to GTR+G, as we used RAxML version 8 (Stamatakis, 2014), which only supports GTR and recommends using the GAMMA approximation for very large matrices. Thus, we were essentially estimating the number of distinct partitions less than or equal to 41 that needed to be modeled using GTRGAMMA in RAxML.

PartitionFinder 2 identified 28 distinct model segments for the GTR-GAMMA model across the 41 possible partitions. We then used ExaML version 3.0 (Kozlov, Aberer & Stamatakis, 2015) to infer phylogenies, under a similar strategy to the one used by Jetz & Pyron (2018). We generated 500 randomized parsimony trees using RaxMLv8.2.9 and used these as starting points for 500 independent ExaML searches. For the highest-scoring ExaML tree, we used the SHL-optimum function in RAxML to perform a final round of NNI-optimization and calculate SHL-support values for nodes, which show strong performance relative to traditional non-parametric bootstrap values (see Pyron et al., 2011). Combined with the newly optimized partitioning strategy and partial re-alignment, this will yield a thorough estimate of the ML phylogeny for this dataset. All data, code, and results are available at Dryad repository DOI 10.5061/dryad.4q3d25m.

### External morphology

The following measurements were obtained from preserved specimens, measured to the nearest 0.1 mm using digital calipers: Snout to Vent Length—SVL, Head Length—HL, Snout Length—SL, Eye Length—EL, Inter Upper Eyelid Width—IUE, maximum Upper Eyelid Width—UEW, Inter-Narial distance—IN, distance between anterior corner of eyes—IFE, Tympanum Diameter—TYD, Fore-Limb Length—FLL, Hind-Limb Length—HAL, First Finger Length—FL1, Second Finger Length—FL2, Third Finger Length—FL3, Fourth Finger Length—FL4, Thigh Length—TL, Crus Length—CL, Foot Length—FOL, Tarsal Length—TAL, Fourth Toe Length—TL4, Inner Toe Length—ITL, Inner Metatarsal Tubercle Length—IMTL, Nostril to Eye Length—NE, Back of Mandible to Nostril Length—MN, Back of Mandible to front of Eye Length—MFE, Back of Mandible to back of Eye Length—MBE, Length between the Back of the Eyes—IBE, Length from Tympanum to the Back of the eye—TE.

### Osteology

High resolution computed tomography (CT) scans were produced for two specimens of the new species, CESF 1565 and CESF 2898, at the GE India Industrial Pvt. Ltd. facility in Pune, India using a Phoenix v|tome|x M (GE’s Measurement & Control business, Boston, MA, USA) with a 180 kV x-ray tube with a diamond-tungsten target and with the following settings: 60–80 kV, 300 mA, a one second detector time, averaging of three images per rotation and a voxel resolution of 3.0–4.9 µm. Additional CT-scans for comparisons were also generated at the Indian Institute of Science or were obtained from MorphoSource, having been generated separately as part of the oVert Thematic Collections Network at either the University of Florida Nanoscale or the University of Chicago PaleoCT lab. Raw x-ray data were processed using GE’s proprietary datos|x software v 2.3 to produce a series of tomogram images that were then viewed and analyzed using VG StudioMax 3.0.3 (Volume Graphics, Heidelberg, Germany). For osteological terminology, we follow Trueb, Diaz & Blackburn (2011), though we refer to the manual digits as I–IV rather than II–V to avoid confusion for most taxonomists. All image stacks, associated metadata, and surface models utilized in this study are available online via MorphoSource (Table S1).

### Taxonomic delimitation

Theoretical and operational criteria for the diagnosis and delimitation of taxa at different ranks are crucial for a robust systematic revision. For diagnosis of species-level taxa, we follow the generalized lineage concept (De Queiroz, 2007) and further split the conventional diagnosis section into lineage diagnosis and field diagnosis (Vijayakumar et al., 2014). The lineage diagnosis provides a description of how the lineage can be diagnosed along multiple axes (phylogenetic, morphological, ecological, and geographic) from related or similar lineages (Shanker, Vijayakumar & Ganeshaiah, 2017). Since there is poor support for sister taxa, we make comparisons with all other relatives within the next well-supported node.

We employed a comprehensive sampling strategy for all frogs, covering the topographic heterogeneity of the WG across latitudinal, elevational and rainfall gradients, ensuring that we detected sympatric relatives with confidence. The field diagnosis illustrates characters that could help diagnose the lineage in the field from syntopic and broadly sympatric species in the sister clades. For higher-level taxa above the species level, we assess whether or not the new lineage occupies a topological position within a currently recognized taxon (genealogical inclusivity), and whether or not it matches the diagnostic characters for that taxon (morphological inclusivity).

The electronic version of this article in Portable Document Format (PDF) will represent a published work according to the International Commission on Zoological Nomenclature (ICZN), and hence the new names contained in the electronic version are effectively published under that Code from the electronic edition alone. This published work and the nomenclatural acts it contains have been registered in ZooBank, the online registration system for the ICZN. The ZooBank LSIDs (Life Science Identifiers) can be resolved and the associated information viewed through any standard web browser by appending the LSID to the prefix http://zoobank.org/. The LSID for this publication is: urn:lsid:zoobank.org:pub:5ADE399C-37CC-4821-BD81-15F420731BB7. The online version of this work is archived and available from the following digital repositories: PeerJ, PubMed Central and CLOCKSS.

## Results

### Molecular phylogeny

**Figure 2:**
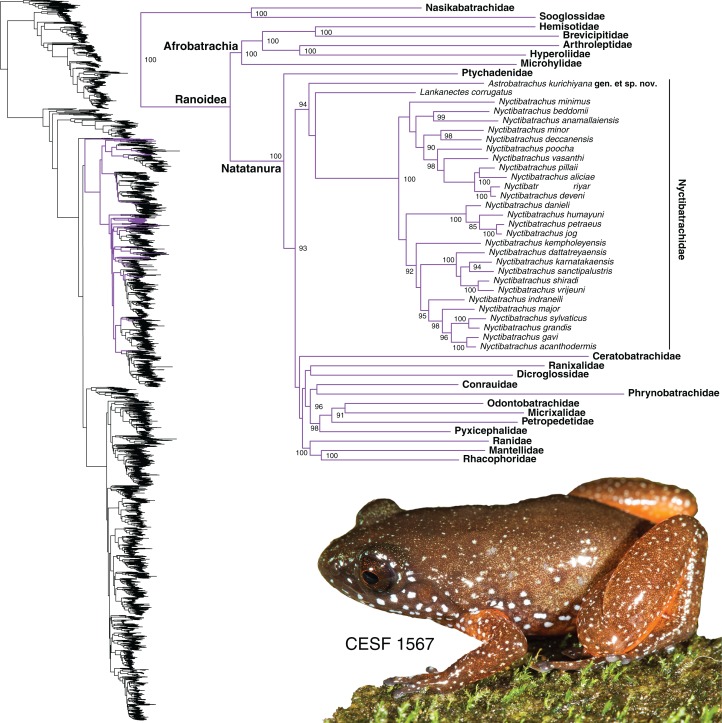
Phylogenetic position of *Astrobatrachus kurichiyana* nested within Natatanura in the clade Nyctibatrachidae. Photo credit: S. P. Vijayakumar.

The unidentified species was found to be a member of the diverse, primarily Old World radiation Ranoidea, specifically within Natatanura (see Feng et al., 2017). It forms a strongly supported clade with the two genera of Nyctibatrachidae (ML, bootstrap support = 94), though the sister relationship between those genera is not strongly supported (Fig. 2). Given the deep divergences between *Nyctibatrachus*, *Lankanectes*, and the new lineage, the age of the MRCA of these three lineages fall within the range of the stem age of Nyctibatrachidae and the crown age of Natatanura, recently estimated to be in the Late Cretaceous or Paleocene (∼57–76 Ma) (Van Bocxlaer et al., 2012; Pyron, 2014; Yuan et al., 2018).

The full phylogeny (see inset on Fig. 2) is provided in the DataDryad repository (see above), and generally agrees with the results of Jetz & Pyron (2018). For the reduced figure (Fig. 2), a random species was chosen from the indicated families. Branch lengths are given in substitutions per million years as estimated from the partitioned GTRGAMMA model in RAxML. Based on the topology and branch lengths from the molecular phylogeny, the unidentified lineage is clearly a highly divergent new species based on genealogical exclusivity. We demonstrate that this new species is also morphologically diagnosable from other natatanuran taxa endemic to the WG. We were thus able to generate both a Lineage and Field diagnosis for a new subfamily, genus, and species. In the following section, we provide the diagnosis and description for the new lineage at each of these taxonomic ranks, in comparison with similar or related taxa.

## Systematics

The lineage shares common ancestry with *Nyctibatrachus* Boulenger, 1882 (a diverse clade composed of >30 species) in the subfamily Nyctibatrachinae, and *Lankanectes* Dubois & Ohler, 2001 (a clade from Sri Lanka with two species) in the subfamily Lankanectinae. It shows deep genetic divergence from these two subfamilies, unique morphological traits (see below; Table 1), and differs in habits, habitat, and distribution from its close relatives (see below). Based on this, we use the phylogenetic position, morphology, terrestrial habits, and geographical distribution to diagnose the subfamily Astrobatrachinae **subfam. nov.** in an expanded Nyctibatrachidae, which is restricted to the WG and Sri Lanka.

**Table 1:** Comparison of morphological characters of Astrobatrachinae, Nyctibatrachinae and Lankanectinae (after Dubois & Ohler, 2001).

	Astrobatrachinae	Nyctibatrachinae	Lankanectinae
Type species	*Astrobatrachus kurichiyana* sp. nov.	*Nyctibatrachus major* Boulenger, 1882	*Lankanectes corrugatus* (Peters, 1863)
Adult male (SVL) in mm	20–27	13–46	33–68.7
Adult female (SVL) in mm	24–29	14–47	44–59
Internarial distance	Equal to distance between upper eyelids	Shorter than distance between upper eyelids	Shorter than distance between upper eyelids
Upper eyelids	Smooth without warts	Without warts or covered with numerous rounded warts	Covered with numerous rounded warts
Canthus rostralis	Distinct	Indistinct or little distinct	Indistinct
Loreal region	Concave	Slightly concave to convex	Slightly convex
Coloration of edge of lower jaw	Without transverse bands	Without transverse bands	Without transverse bands
Tympanum	Distinct	Indistinct or little distinct	Indistinct
Extremities of digits	Mostly triangular without dorsoterminal folds	Disks bearing dorsoterminal folds	Extremities of fingers pointed, or toes slightly rounded without dorsoterminal folds
Relative length of fingers one and two	Mostly sub-equal	Finger two longer than finger one	Indistinct
Distal sub articular tubercles on finger three and four	Indistinct and primitive	Indistinct	Absent
Inner palmar tubercle	Absent	Small, oval rather prominent, on base of metacarpus	Very small, rounded on base of metacarpus
Outer palmar tubercle	Absent	Oval, about half size of inner palmar tubercle	Very small, rounded, of same size as inner palmar tubercle
Hind legs	Weak and short	Strong, short	Very strong, short
Distance between heels when hind legs are placed at right angle with body	Heels just touch	Heels far apart	Heels far apart
Tarsal fold or ridge	Absent	Present well developed	Present well developed
Inner metatarsal tubercle	Present, primitive (no distinction between inner metatarsal tubercle and sub articular tubercle)	Long, oval prominent	Flat, elongate
Tarsal tubercle	Absent	Absent	Absent
Femoral glands	Presence doubtful, appears faint in life, not raised on or below the skin	Present/Absent	Present/Absent
Subocular gland	Absent	Present	Absent
Lateral line system in adult	Absent	Absent	Present
Network of ridges on back and head	Absent	Absent	Present
Coloration of rear part of thighs	No pattern, cream white with faint minute dotted gray marbling	Marbled	Marbled
Sex size dimorphism	Absent	Absent	Absent
Fangs in adult male	Absent	Absent	Present
Vocal sacs in adult male	NA	Present, internal, with folds on throat	Present, internal, without folds on throat
Male advertisement call	NA	Present	Present
Nuptial pads in adult male	Absent	Present	Absent/Present
Amplectic position	NA	Absence of physical contact	Axillary amplexus
Egg coloration	NA	Pigmented or not	Pigmented
Mode of development	NA	Tadpole	Tadpole
Parental care	NA	Present/Absent	Absent
Eye color pattern	Oval, black pupil with bright golden orange ring; iris brownish with interspersed golden yellow sparks	Mostly pupil rhomboidal with golden brown iris	Pupil rhomboidal with golden brown iris
Digital tips shape	Mostly triangular	Blunt rounded to enlarged discs	Rounded, enlarged without disc
Dorsoterminal grooves	Absent	Discs with or without dorsoterminal grooves	Absent
Dorsal skin pattern	Smooth, glandular	Shagreened, glandular or wrinkled skin	Corrugated with transverse skin folds
Webbing between fingers	Dorsally rudimentary between fingers three and four; ventrally rudimentary	Absent	Absent
Median lingual process	Absent	Present	Absent
Dorsal color pattern	Brown, with bluish spots that are prominent on the lateral side	Dark brown to light brown with or without patterns	Brown to chocolate brown
Ventral color pattern	Light gray with bluish spots on the throat region; bright orange at the region of stomach and thighs	Varying from cream white to interspersed brown spots	White mottled with brown
Species contained	Monotypic	36 species (Frost, 2018)	2 species (Senevirathne et al., 2018)
Geographical distribution	Western Ghats, Peninsular India	Western Ghats, Peninsular India	Sri Lanka
		Dubois & Ohler, 2001; Biju et al., 2011	Dubois & Ohler, 2001; Biju et al., 2011; Senevirathne et al., 2018

Amphibia Linnaeus, 1758
Anura Fischer von Waldheim, 1813
Ranoidea Batsch, 1796
Natatanura Frost et al., 2006
Nyctibatrachidae Blommers-Schlosser, 1993
Astrobatrachinae **subfam. nov.** urn:lsid:zoobank.org:act:28F98D95-3E1E-41BB-B7A1-CC6766432E22

**Type genus.—***Astrobatrachus* **gen. nov.** urn:lsid:zoobank.org:act:28F98D95-3E1E-41BB-B7A1-CC6766432E22, by present designation.

**Etymology of the generic nomen.—**From the Greek *astro*- for ‘star,’ referring to the starry spots, more prominent on the lateral sides of the body, and *batrachus* meaning ‘frog’. As per the nomenclatural act the gender of genus is ‘male.’

**Type species.—***Astrobatrachus kurichiyana* **sp. nov.** urn:lsid:zoobank.org:act:A4A21F51-2A11-41C6-AA2A-B6F390070868, by present designation

**Figure 3:**
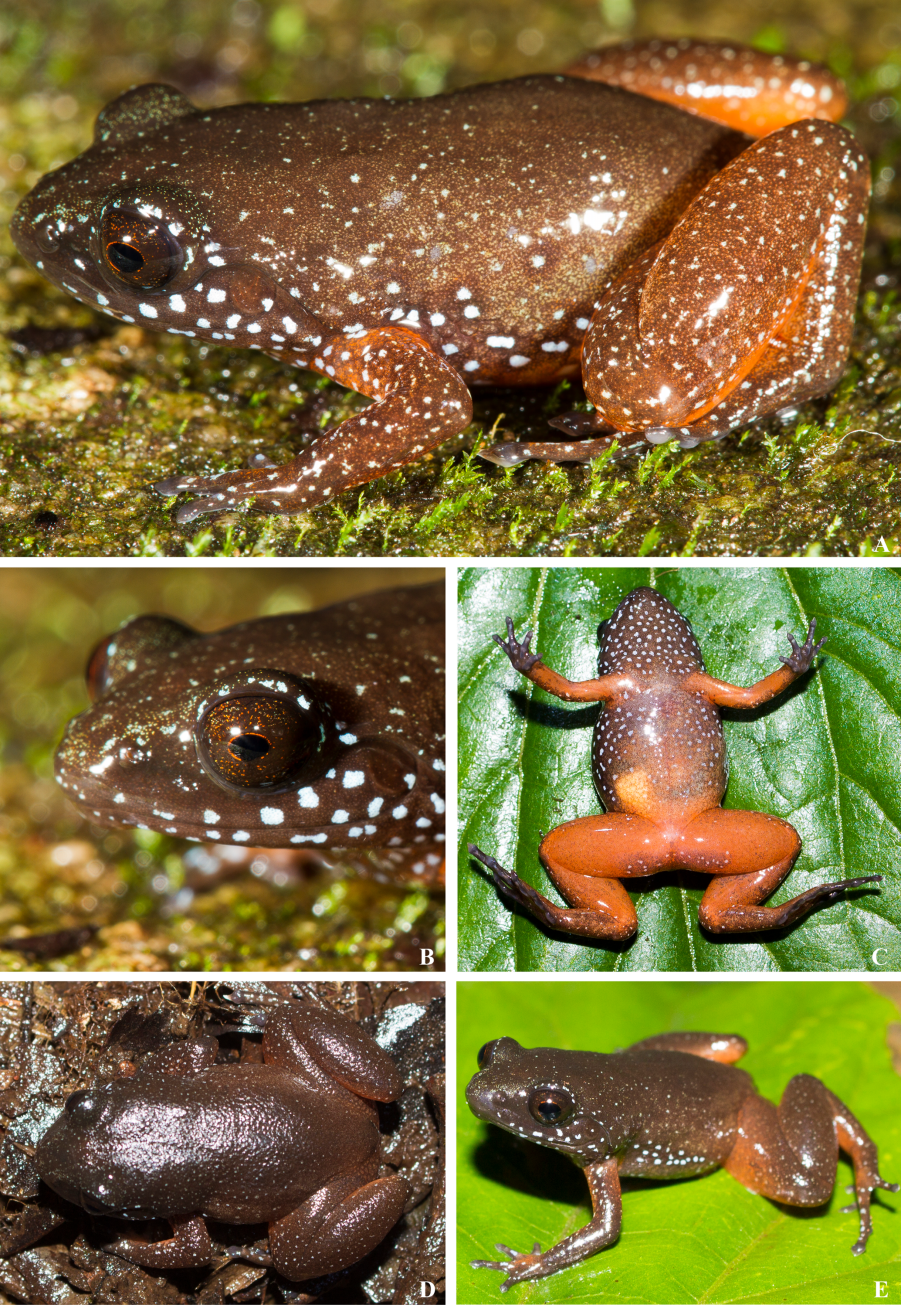
Live images of *Astrobatrachus kurichiyana*. Profile (A), close-up of head (B), ventral (C), dorsal (D), side-profile (E). Photo Credit: S.P. Vijayakumar (A and B; reference collection CESF 1567), K.P. Dinesh (C, D and E; ZSI/WRC/A/2131)

**Holotype.—**ZSI/WRC/A/2131 (male, Fig. 3C–3E) by present designation, collected by S.P. Vijayakumar, K.P. Dinesh, and a team from Kurichiyarmala (11.602807° N 75.968025° E, alt.1420 m), Wayanad Plateau, WG during an expedition on 21st June 2010.

**Paratypes.—**ZSI/WRC/A/2132 (CESF 1565) (male), and ZSI/WRC/A/2133 (CESF 2897) (female); site same as holotype collection.

**Figure 4:**
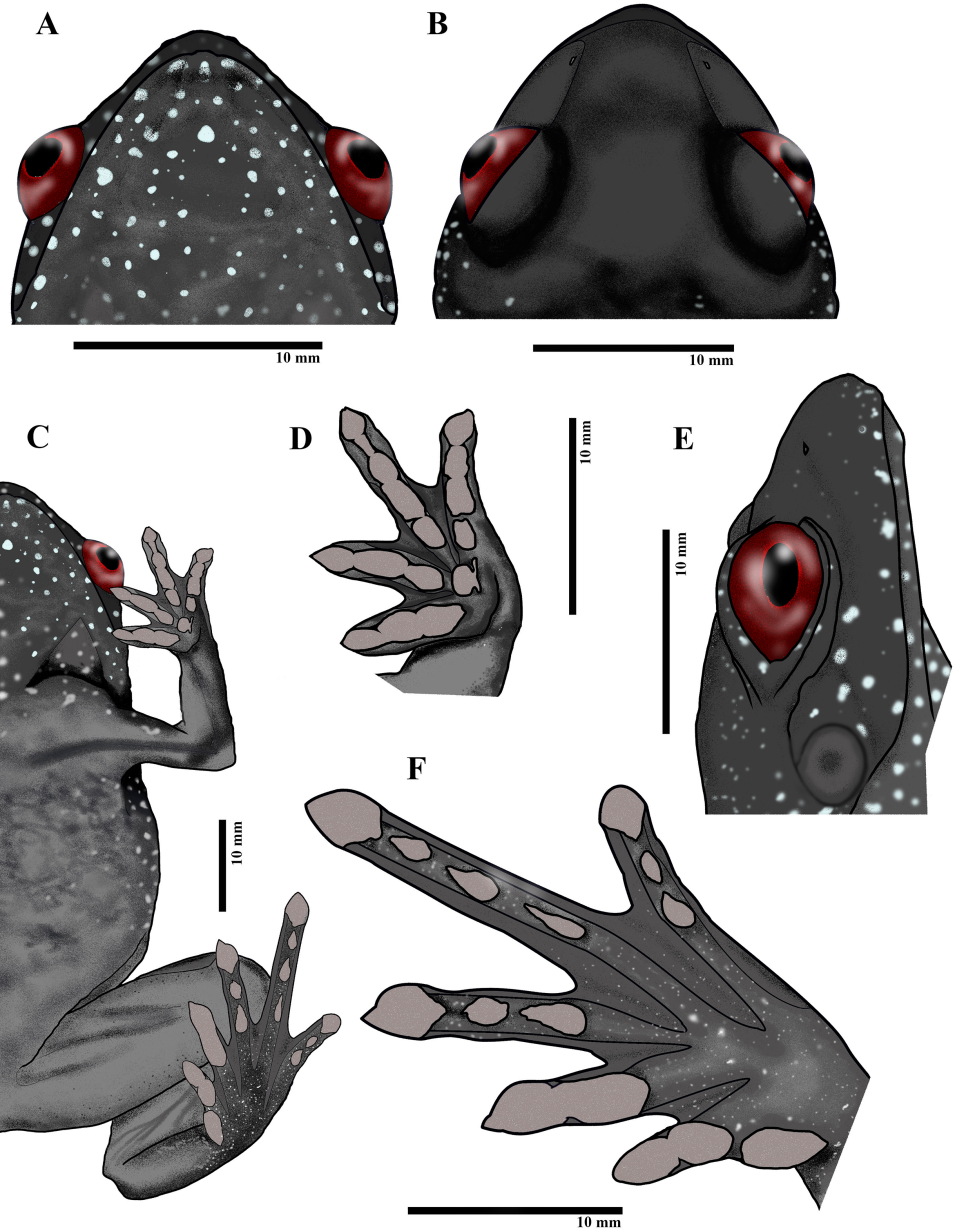
Paratype of *Astrobatrachus kurichiyana*- ZSI/WRC/A/2133 (female): Ventral head (A), dorsal head (B), ventral (C), ventral fingers (D), lateral head (E), ventral toes (F). Drawing Credit: Achyuthan N. Srikanthan

**Diagnosis.—**This diagnosis applies to the subfamily, genus, and species. The following combination of characters can be used to diagnose this lineage from its close relatives *Nyctibatrachus* and *Lankanectes*: small to medium size (∼ 20–27 mm SVL); soft skin without ridged or wrinkled folds; fingers and toe tips with discs that are triangular in shape (Figs. 3 and 4) without circummarginal groove; upper jaws having distinct teeth; distinct and angular canthus rostralis; distinct tympanum with a prominent supra-tympanic ridge (Fig. 3); tongue lacking median papilla; short hind and fore-limbs; oblong subarticular tubercles on the fingers and toes that sometimes nearly coalesce (e.g., pedal digit III in Figs. 3 and 4); interdigital webbing on foot does not attain most proximal subarticular tubercle; absence of femoral glands; absence of nuptial pads in males; widely spaced nasal bones; a vomer separated into an anterior portion adjacent to the choana and a posterior dentigerous vomer fused to a neopalatine; omosternum not bifurcating posteriorly; a single narrow sternal element; lacking a large dorsal crest on the ilium; bluish-white spots (Figs. 3 and 4), more prominent and scattered along the lateral sides of jaws, eyelids, belly, forearms and hind limbs, and on the throat; oval-shaped pupil; orange coloration of ventral sides of belly, forelimbs and hind limbs; elliptical pupil (Fig. 3). The lineage is diagnosed easily in the field from species of *Nyctibatrachus* that occur sympatrically.

### Description of holotype ZSI/WRC/A/2131 (male)

A small-sized frog (SVL = 20.2 mm) with a squat, lean body (see Table 2); head length approximately equal to head width (HL = 7.2 mm; HW = 7.3 mm); snout acutely pointed in dorsal and obtusely pointed in ventral views; snout length (SL = 3.0 mm) approximately equal to eye diameter (EL = 2.9 mm); canthus rostralis angular, loreal region concave; inter orbital space flat and wide (IUE = 2.8 mm), 2.3 times more than upper eye lid (UEW = 1.2 mm) and subequal to inter-narial distance (IN = 2.6 mm); distance between back of eyes two times more than front of eyes (IFE = 3.8 mm; IBE = 7.8 mm); nostrils oval, nearer to tip of snout and lateral in position; symphysial knob weak; tympanum distinct visible below supratympanic fold (TYD = 1.2 mm); weak vomerine ridges present with three to four teeth; tongue bifid without papilla; lower jaw without teeth, upper jaw with distinct sharp teeth. Forearm slender (FLL = 5.8 mm) longer than hand (HAL = 4.0 mm); fingers short and thin without dermal fringes; relative finger length IV < I < II < III (FL1 = 2.3 mm, FL2 = 2.8 mm, FL3 = 3.4 mm, FL4 = 2.0 mm); finger tips triangular and blunt without circummarginal groove; webbing between fingers rudimentary; subarticular tubercles of fingers oblong with indistinct margins; pollex tubercle absent; supernumerary tubercles absent. Hindlimbs short, just touching when adpressed, and tibio-tarsal articulation reaches back of eyes; thigh length approximately equal to crus length (TL = 9.3 mm; CL = 9.0 mm); foot length is greater than tarsus length (FOL = 9.3 mm, TAL = 6.8 mm), relative toe length I < II < V < III < IV (TL4 = 4.8 mm; ITL = 2.0 mm); webbing rudimentary (not reaching the most proximal subarticular tubercle); inner metatarsal tubercle (IMTL = 1.0) small and nearly continuous with subarticular tubercle of first toe; subarticular tubercles of toes oblong and indistinct, coalescing on some toes; outer metatarsal tubercle absent and supernumerary tubercles absent. Overall skin on dorsum soft, finely glandular, ventrally smooth glandular. Rictal gland and ocular gland absent.

**Table 2:** Morphometric data for *Astrobatrachus kurichiyana*.

	ZSI/WRC/A/2131 (Holotype)	ZSI/WRC/A/2132 (CESF 1565) (Paratype)	ZSI/WRC/A/2133 (CESF 2897) (Paratype)
Sex	M	M	F
SVL	20.2	27.3	27.2
HW	7.3	9.2	9.4
HL	7.2	9.3	8.9
IN	2.6	3.2	3.2
NE	1.6	2.0	1.8
MN	6.0	7.2	7.2
MFE	4.9	6.1	5.9
MBE	2.2	2.7	2.8
SL	3.0	4.2	4.0
EL	2.9	4.0	4.0
IUE	2.8	3.7	3.8
UEW	1.2	2.0	1.8
IFE	3.8	5.0	4.8
IBE	7.8	8.6	8.4
TYD	1.2	1.8	1.8
TE	1.0	1.4	1.4
FLL	5.8	6.8	6.6
HAL	4.0	5.5	5.1
FL1	2.3	2.8	2.8
FL2	2.8	3.1	3.2
FL3	3.4	3.8	3.8
FL4	2.0	2.1	2.1
TL	9.3	12.2	12.2
CL	9.0	12.0	12.0
TAL	6.8	8.2	8.2
FOL	9.3	11.0	10.8
TL4	4.8	6.4	6.2
ITL	2.0	2.6	2.6
IMT	1.0	1.4	1.2

### Coloration of holotype

In life, overall color on dorsum rufous brown, darker in anterior region of body and light in posterior part of body. Small pale bluish-white spots present all over body but larger and more concentrated on lateral, gular, anterior ventrum, and around tympanic region, jaws, and eyelid. In ventral region, arm, belly, thigh, and crus bright orange in color; gular region is dark gray with bluish white spots. Under surface of hand and foot dark gray. In preservative, color on dorsum dark brown with cream spots. Gular and anterior ventrum dark gray interspersed with cream spots, with melanocytes becoming widely spaced posteriorly; arm, posterior belly, thigh, and crus cream colored; hand and foot dark gray with pale gray skin under digit tips and subarticular tubercles.

### Osteology

**Figure 5:**
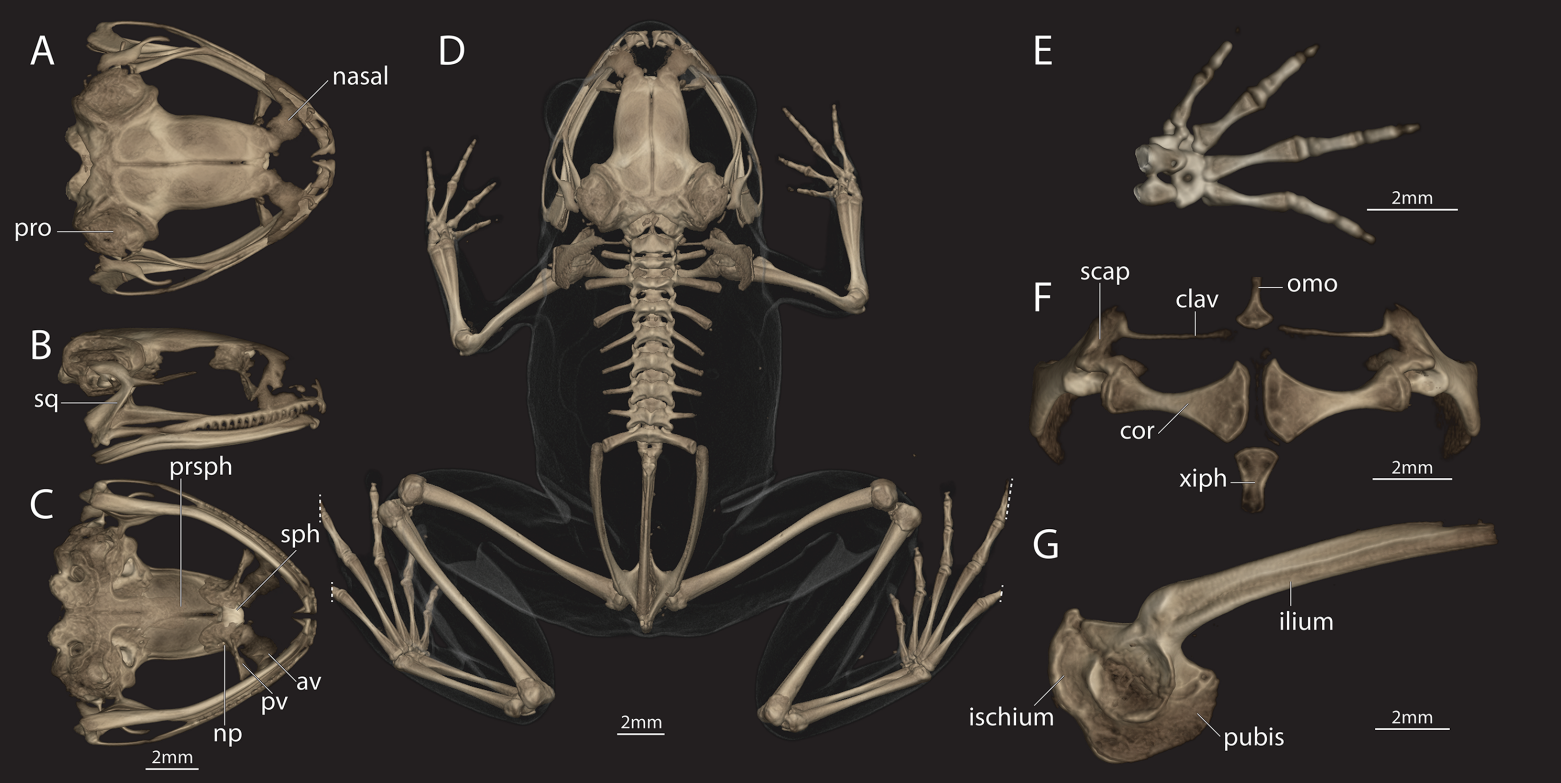
Female of *Astrobatrachus kurichiyana* (reference collection CESF 2898). Female of *Astrobatrachus kurichiyana* (CESF 2898) as visualized via microCT-scanning with skull in dorsal (A), right lateral (B), and ventral (C) views, entire skeleton in dorsal view (D), right hand in dorsal view (E), pectoral girdle in ventral view (F), and pelvis in right lateral view (G). Note that distal extent of the fourth and fifth toes on both feet were truncated during CT-scanning. Abbreviations of anatomical terms are as follows: av, anterior vomer; clav, clavicle; cor, coracoid; np, neopalatine; omo, omosternum; pro, prootic; prsph, parasphenoid; pv, posterior vomer; scap, scapula; sph, sphenethmoid; sq, squamosal; xiph, xiphisternum.

**Figure 6:**
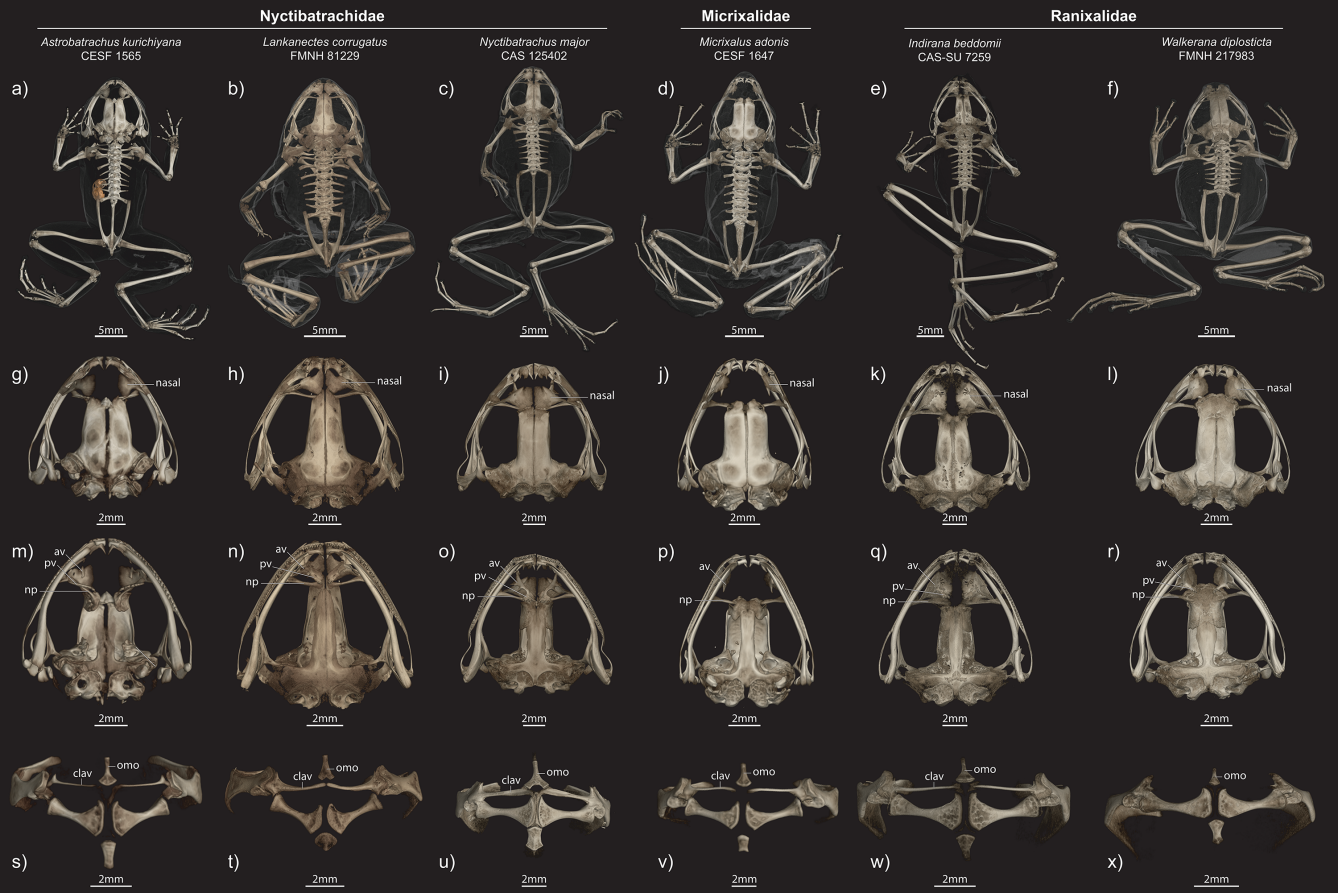
Comparisons of male paratype (ZSI/WRC/2132) of *Astrobatrachus kurichiyana* to representatives of other WG endemics. Exemplars from Nyctibatrachidae, Micrixalidae, and Ranixalidae in different views ((A–F) entire skeleton in dorsal view; (G–I) dorsal view of skull; (M–R) ventral view of skull; (S–X) ventral view of pectoral girdle). Abbreviations of anatomical terms are as follows: av, anterior vomer; clav, clavicle; np, neopalatine; omo, omosternum; pv, posterior vomer.

Based on micro-CT scan of ZSI/WRC/2132 (paratype; male) and CESF 2898 and 2899 (reference collections, females) (Figs. 5 and 6). The skull is lightly constructed, slightly longer than wide, lacking ornamented dermal bones, and having jaw joints that are anterior to the occiput. The prootics are incompletely ossified and not synostosed to the exoccipital or frontoparietals. The lateral walls of the neurocranium are not ossified such that the optic fenestrae and prootic foramina are not bounded in bone. The frontoparietals are broad, approximately 3/5 the width of the length, and do not articulate at the midline. The nasals are widely spaced. The premaxillae bear teeth, and each has a prominent pars dentalis and a robust alary process that is taller than wide and widely separated from the nasal. The maxillae bear teeth for approximately two-thirds of their length and are nearly straight. The quadratojugals are elongate, extending anterior to the body of the squamosal. The pterygoids are triradiate, each with a long anterior ramus that articulates with the maxilla near the posterior level of the maxillary teeth, a long posterior ramus extending to the jaw joint, and a short and thin medial ramus extending towards but not articulating with the prootic. The anterior portion of the vomer is small and distinct, forming a C-shape around the anteromedial surface of the choanae. This anterior portion is not connected to the dentigerous posterior portion that is synostosed to the neopalatine. The sphenethmoid is located anterior to the rostral extent of the parasphenoid, either abuts or is synostosed to the neopalatine, and has well-ossified tectum and septum nasi that separate prominent circular olfactory foramina. The parasphenoid is triradiate with the rostral extent of the cultriform process being V-shaped and not attaining the sphenethmoids, and the lateral alae underlying the otic capsule. The squamosals are prominent with a zygomatic ramus that tapers rostrally, and a posterior ramus forming a small shelf just lateral to, but not synostosed to, the proootic. A thin distally tapering stapes (or columella) is present, terminating just below the posterior ramus of the squamosal; an ossified operculum is not present. The posteromedial processes of the hyoid are short and weakly expanded at their posterior ends.

There are eight distinct, procoelous, non-imbricating presacral vertebrae that are not fused to one another and all of which have a large neural canal. The atlas lacks transverse processes and has widely separated cotyles. The transverse processes of Presacrals II are directly laterally, whereas those of Presacrals III–VII are directed posteriorly. Presacral VIII has weakly anteriorly directed transverse processes and a biconvex centrum, making the vertebral column displasiocoelous. The sacrum is procoelous with short transverse processes and has two condyles posteriorly. The urostyle bears two cotyles, which are not confluent, for articulation with the urostyle and a tall dorsal ridge that tapers posteriorly, ending approximately at the anteroposterior midpoint.

The pectoral girdle is firmisternal and no elements are in direct articulation or synostosed. The scapula has a defined waist, expanding dorsally, and has an expanded and rounded acromial process. The clavicle is thin and nearly perfectly straight. The coracoid is widely expanded medially, with the medial extent approximately twice as wide as the lateral extent. A small omosternum is expanded posteriorly and tapers anteriorly. A stout and nearly rectangular bony xiphisternum projects distally from the coracoids.

In CESF 2898, the three elements of the pelvic girdle—ilium, ischium, and pubis— are ossified and synostosed to one another. The shaft of the ilium is long and bears a weakly developed supraacetabular fossa, a well-defined dorsal protuberance, and a short dorsal crest for most of its length. The synostosis of the pelvic elements creates a broad sheet of bone posterior, ventral, and anterior to the acetabulum. In CESF 1565, the ventral acetabular expansion projects anteriorly, but not ventrally below the point at the margin of the acetabulum at which the ilium and pubis meet; the ischium and pubis are not fully ossified in this specimen.

The humerus is thin and straight with a weakly developed ventral crest and entepicondyle. The forearm is approximately two-thirds the length of the humerus. The radiale and ulnare are large and elongate, and subequal in size. The distal carpals are fused into two groups: Element Y + distal carpal 2, and distal carpals 3+4+5. The phalangeal formula for the manus is 2–2–3–3 and there are two small ossified elements of the prepollex that are more ossified and discernable in the larger specimens (CESF 2898 and 2899). The terminal manual phalanges are blunt distally, and in some cases tapering. The tibiofibula and femur are similar in length. There is a tarsal sesamoid on the ventral surface at the articulation of the tibiofibula and the tibiale and fibulare. There are two distal tarsals, two small ossified elements in the prehallux, and a sesamoid on the plantar surface at the base of metatarsals IV and V. The phalangeal formula for the pes is 2–2–3–4–3. The terminal pedal phalanges taper and then expand into a small blunt tip.

### Comparisons

*Astrobatrachus* is clearly distinguishable from other members of the Nyctibatrachidae as well as other families endemic to the WG (Table 3). No information is available on the larval biology or reproductive modes of *Astrobatrachus*. *Astrobatrachus* differs from *Lankanectes* in lacking dorsal skin ridges, lacking a lateral line system as an adult, having a distinct tympanum and canthus rostralis, lacking odontoid fangs, having widely spaced nasals, having a long ridge of vomerine teeth, having separate anterior and posterior portions of the vomer with the latter fused to the neopalatine, lacking a forked omosternum, lacking a prominent dorsal crest on the ilium, and lacking both a tarsal fold and webbed pedal phalanges (Dubois & Ohler, 2001; Senevirathne et al., 2018; Figs. 5 and 6). Similar to *Lankanectes*, *Astrobatrachus* lacks a median lingual papilla and femoral glands, lacks circummarginal grooves on the digits (present in some *Nyctibatrachus*), has a second finger longer than the first finger, and indistinct subarticular tubercles.

**Table 3:** Morphological character differences between *Astrobatrachus kurichiyana*and the potentially sympatric or syntopic relatives of *Nyctibatrachus*.

Characters/species	*N. grandis*	*N. minimus*	*N. vrijueni*	*N. kempholeyensis*	*N. indraneli*	*Astrobatarchus kurichiyana*
Size (mm)	62.2–76.9	10.0–13.5	38.7–43.1	15.5–21.6	42.5	20.2–27.2
Skin	Wrinkled with granular projections	Interrupted dorsolateral folds and faint granular projections	Wrinkled skin	Wrinkled skin with granular projections	Wrinkled	Smooth
Webbing between fingers	Absent	Absent	Absent	Absent	Absent	Present, rudimentary
Webbing between toes	Webbed/extends beyond 3rd subarticular tubercle	No webbing	Webbed/barely reaching the 3rd subarticular tubercle	Webbed/reaching above 2nd subartcular tubercle	Webbing reaching beyond 3rd subarticular tubercle	Rudimentary
Dorsoterminal groove on fourth toe disc	Present, cover rounded distally	Present, cover bifurcate distally	Present, cover notched distally	Present, cover rounded distally	Absent	Absent
Subarticular tubercles	Prominent, oval,	Prominent, rounded	Prominent, oval	Prominent, oval	Prominent, oval	Primitive, glandular
Toe discs	Well developed	Weakly developed	Well developed	Well developed	Well developed	Triangular shaped
Coloration	Dorsum uniform dark gray with light gray patches	Dorsum tan without prominent patterns, scattered minute white spots on lateral side	Uniform reddish-brown	Light brownish-gray	Grayish brown with yellow patches	Brown, with bluish spots that are prominent on the lateral side
Tympanum	Indistinct	Indistinct	Indistinct	Indistinct	Indistinct	Distinct
Femoral glands	Present	Present	Present	Present	Present	Absent
Subocular gland (proposed by Biju et al., 2011)	Present	Present	Present	Present	Present	Absent
Tarsal fold	Tarsal fold present	Tarsal fold present	Tarsal fold present	Tarsal fold present	Tarsal fold present	Absent
Black or bluish-black liver—visible on the ventral side through skin in life	Yes	Yes	Yes	Yes	Yes	No
Ridge extending from the lip over the tip of the snout to between the nostrils	Present	Absent	Present	Present	Present	Absent

*Astrobatrachus* differs from *Nyctibatrachus* in lacking rugose or wrinkled skin with longitudinal folds, having a distinct tympanum and canthus rostralis, lacking a subocular gland, having widely spaced nasals, having a long ridge of vomerine teeth, having separate anterior and posterior portions of the vomer with the latter fused to the neopalatine, lacking a forked omosternum, lacking femoral glands, lacking a prominent dorsal crest on the ilium, lacking both a tarsal fold and webbed pedal phalanges, lacking a rhomboidal pupil (Biju et al., 2011; Chandramouli & Dutta, 2015). Similar to *Nyctibatrachus*, *Astrobatrachus* lacks a median lingual papilla (present in some *Nyctibatrachus*), lacks a lateral line system as an adult, and has a second finger longer than the first finger and indistinct subarticular tubercles.

The skeleton of *Astrobatrachus* is also distinguishable from genera of Micrixalidae (*Micrixalus*) and Ranixalidae (*Indirana*, *Walkerana*). *Astrobatrachus* differs from *Micrixalus* by having vomerine teeth and a posterior portion of the vomer, and lacking T- or Y-shaped terminal phalanges (Dubois, 1986), but is similar by having widely spaced nasals, an unforked omosternum, and thin, straight clavicles. *Astrobatrachus* differs from *Indirana* by having widely spaced nasals, having a separate posterior vomer fused to the neopalatine, lacking a T- and Y-shaped terminal phalanges, but is similar by having an unforked omosternum, and thin, straight clavicles. *Astrobatrachus* differs from *Walkerana* by having widely spaced nasals, a separate posterior vomer fused to the neopalatine, a clavicle (absent in *Walkerana*), a well-defined sternum, and lacking Y-shaped terminal phalanges, but is similar by having an unforked omosternum.

### Habits and habitat

**Figure 7:**
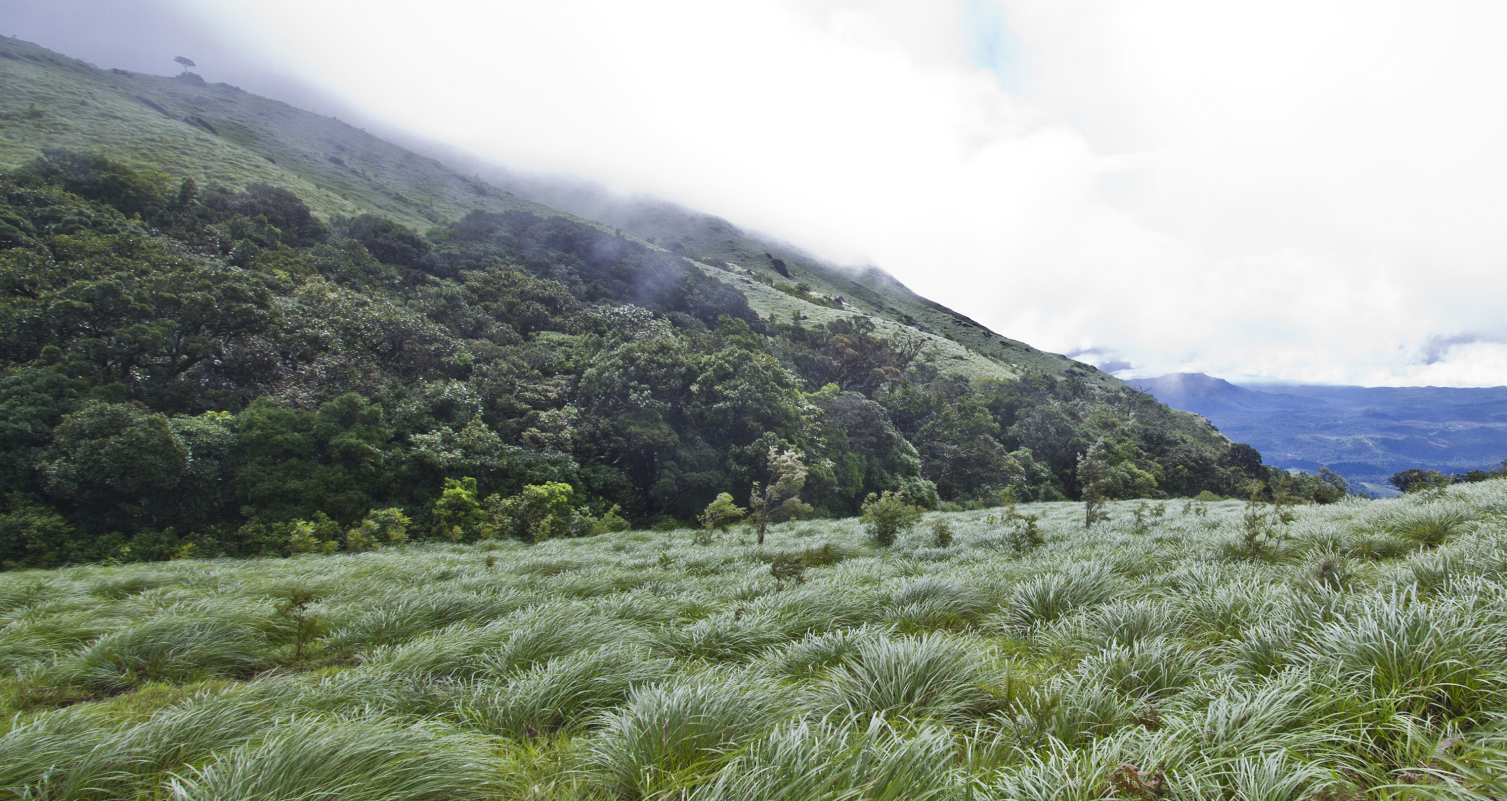
Type locality of *Astrobatrachus kurichiyana*. Most individuals were sighted in the montane forests except for a single individual in the grassland. Locality: Kurichiyarmala, Wayanad Plateau. Photo taken: June 2010. Photo Credit: S.P. Vijayakumar.

The new species is nocturnal and found below decayed leaf litter within montane forests in the vicinity of water. One individual was caught moving in a grassland adjoining the forest tract (Fig. 7). On the forest floor, where most individuals were sampled, they hid under leaf litter when disturbed. Because individuals were secretive and difficult to spot, sampling involved an intensive search of the forest floor. Individuals were found to be shy of torch light and upon disturbance, made quick hopping movements to hide. No individuals were found exposed during the night during either rainy or non-rainy periods. As a general observation, most sympatric anurans in the region usually emerge in the dark and call during the rain or post rain seasons. Leaf-litter dwelling and habitat distinguishes *A. kurichiyana* from many species of *Nyctibatrachus* that are torrential frogs and prefer to live in water or next to perennial streams (Biju et al., 2011). While its terrestrial habits are somewhat similar to some small-bodied *Nyctibatrachus* species (see Garg et al., 2017), the new lineage differs strongly from the two *Lankanectes* species which are aquatic (Senevirathne et al., 2018).

### Distribution

All known populations of this species occur in Kurichiyarmala on the Wayanad Plateau, in the WG Escarpment (Fig. 1). The geographical range of Nyctibatrachinae, widespread across the WG, overlaps with Astrobatrachinae (Fig. 1). However, both lineages have a disjunct distribution with respect to Lankanectinae, which is restricted to the mountains of Sri Lanka (Fig. 1). The new species occurs in syntopy and in broad sympatry with *Nyctibatrachus grandis*, *N. minimus*, *N. vrijeuni*, and *N. kempholeyensis*.

### Etymology

From ‘Kurichiyana,’ a local tribal community residing near the type locality and currently known geographic range of the species. Species epithet is treated as a noun in apposition to the generic name. We suggest the common English name of the Starry Dwarf Frog.

### Conservation status

Based on the lack of information on the abundance of the species, and inadequate data on the geographical range of the lineages, using the IUCN Red List criteria, we suggest *A. kurichiyana* be assigned to the Data Deficient category.

## Discussion

Here, we report a new, deeply divergent, monotypic lineage, recognized as a new subfamily Astrobatrachinae within Nyctibatrachidae, and a new genus and species *A. kurichiyana*. Since this lineage forms a well-supported clade with Nyctibatrachinae and Lankanectinae, but is highly divergent both morphologically, including osteology, and genetically, we erect a new subfamily—Astrobatrachinae—endemic to the WG of Peninsular India. A sister relationship between *Nyctibatrachus* and *Lankanectes* has long been supported by molecular phylogenetic analyses (Vences et al., 2000; Roelants, Jiang & Bossuyt, 2004; van der Meijden, Vences & Meyer, 2004; Delorme et al., 2004; Bossuyt et al., 2006). However, with the addition of this new lineage, the relationship among the three subfamilies becomes uncertain. While we place Astrobatrachinae as a subfamily in Nyctibatrachidae as a matter of taxonomic conservatism, we note that the ancient divergence and lack of unifying morphological synapomorphies may support recognition of Astrobatrachidae as a distinct family in the future.

The relationships of Nyctibatrachidae with other WG endemic families such as Micrixalidae and Ranixalidae have also been unstable across analyses, typically with low support. Sometimes Micrixalidae and Ranixalidae are sister lineages (Roelants, Jiang & Bossuyt, 2004; Bossuyt et al., 2006), sometimes Micrixalidae and Nyctibatrachidae are (Roelants et al., 2007), and sometimes none of these forms a clade (Frost et al., 2006; Pyron & Wiens, 2011). Bossuyt & Milinkovitch (2000, 2001) and later Roelants, Jiang & Bossuyt (2004) provided the first indication that these three natatanuran families may be among the earliest diverging in a larger clade of Malagasy and Asian families.

Recently, Yuan et al. (2018) published a well-supported phylogenomic analysis in which Ranixalidae and Nyctibatrachidae were found to form a clade sister to other Asian and Malagasy natatanurans: Ranidae, Rhacophoridae, Mantellidae, Ceratobatrachidae, and Dicroglossidae. At present, there are no clear morphological synapomorphies supporting the monophyly of Nyctibatrachidae with respect to other natatanurans. However, this is in part due to the lack of a clear phylogenetic context for the relationships of this family. A robust phylogenomic analysis containing all of the natatanuran families, including the three endemic WG families, would help to then make comparisons among families to determine distinguishing characteristics of Nyctibatrachidae within a subclade.

Under our new arrangement, Nyctibatrachidae is a diverse clade containing three divergent subfamilies each with unique morphology and ecology. More than two-thirds of the recognized nyctibatrachid species have been described in the past two decades (25 of 36 in *Nyctibatrachus* Boulenger, 1882, and one of two in *Lankanectes* Dubois & Ohler, 2001). Similarly, two other natatanuran families endemic to the WG have also seen many new species described during this time (8 of 14 species of *Indirana* Laurent, 1986, family Ranixalidae; 14 of 24 species of *Micrixalus* Boulenger, 1888, family Micrixalidae).

The genus *Nyctibatrachus* contains predominantly torrent-dwelling species (Biju et al., 2011; Garg et al., 2017). The Sri Lankan *Lankanectes* is almost entirely aquatic, but resides in slower-moving streams (Delorme et al., 2004; Senevirathne et al., 2018). The only known species of *Astrobatrachus* comprises entirely terrestrial forest-dwellers that prefer leaf-litter habitats. Like *Astrobatrachus*, a few species such as *N. minimus* (discovered in the same hill range as *A. kurichiyana*) are occasional litter-dwellers (Biju et al., 2007; Garg et al., 2017). However, *A. kurichiyana* is restricted to forest floor litter, unlike most syntopic nyctibatrachids. No information is yet available on the larval biology or reproductive modes of *Astrobatrachus*.

### Ancient lineages: signatures of a tropical mountain refuge

The discovery of this lineage adds to the growing list of higher taxa endemic to the WG including *Beddomixalus* Abraham, Pyron, Ansil, Zachariah, and Zachariah, 2013; *Ghatixalus* Biju, Roelants, and Bossuyt, 2008; *Ghatophryne* Biju, Van Bocxlaer, Giri, Loader, and Bossuyt, 2009; *Indirana; Melanobatrachus* Beddome, 1878; *Mercurana* Abraham, Pyron, Ansil, Zachariah, and Zachariah, 2013; *Micrixalus; Nasikabatrachus* Biju & Bossuyt, 2003; *Nyctibatrachus; Pedostibes* Günther, 1876; *Walkerana* Dhanaukar, Modak, Krutha, Nameer, Padhye, and Molur, 2016; and *Xanthophryne* Biju, Van Bocxlaer, Giri, Loader, and Bossuyt, 2009 (see Biju, Roelants & Bossuyt, 2008; Biju et al., 2009; Abraham et al., 2013; Dahanukar et al., 2016). Many of these lineages—except *Indirana*, *Nyctibatrachus* and *Micrixalus*—are also relatively depauperate, having only one or two species. In contrast, a number of their close relatives, which originated within a similar time period, are species rich (e.g., *Nyctibatrachus* >30 species) which suggests either the failure of some lineages to diversify, incomplete sampling, or extinctions over time. At this juncture, our study and other extensive surveys exclude poor sampling as a cause. The peculiar mixture of hyperdiverse radiations such as *Pseudophilautus* Laurent 1943 and *Raorchestes* Biju, Shouche, Dubois, Dutta, and Bossuyt, 2010 (see Meegaskumbura et al., 2002; Vijayakumar et al., 2016) along with the numerous ancient and species-poor microendemics remains an unexplained evolutionary phenomenon.

Barring *Xanthophryne*, a lineage distributed in the northern plateau of the WG, all the recently recognized species poor genera, two of the sub-families (Astrobatrachinae and Lankanectinae) and the family Nasikabatrachidae have narrow ranges and are restricted to different massifs in the southern and central latitudes of the Escarpment and Sri Lanka. The southern regions of the WG (and Sri Lanka) have long been considered ancient climatic refuges (Mani, 1974), and recent discoveries of ancient lineages from these southern massifs underscores their evolutionary significance. Recent palynological research has indicated presence of a tropical rainforest refugium in the lowlands of the southern WG (Prasad et al., 2009) but the extent and location of the proposed refugia in these southern regions is unclear. Age estimates of the MRCA suggest that lineages range from the Late Cretaceous to Miocene. The discovery of this new lineage provides further impetus towards testing alternative hypotheses driving endemism and diversification in clades in this biodiversity hotspot.

*Astrobatrachus kurichiyana* is distributed at high elevations (1,300–1,400 m) of a small massif in the WG escarpment (Fig. 1). Its distribution needs to be assessed in the poorly explored adjoining massifs Vellarimala (Camel’s Hump) and Banasuramala to further confirm its microendemic status. While we are not aware of imminent threats to this ancient lineage, additional surveys are needed to quantify its geographical range, population density and habitat specificity to determine if conservation measures are required. Considering its age and the possibility of a narrow range, and its location outside protected areas, the significance of reserve forest tracts in the WG in housing evolutionary novelties needs to be highlighted. Frequent discoveries of older lineages from the southern and central massifs of the WG suggest that additional intensive surveys in these massifs are bound to uncover more lineages and advance our understanding of the historical biogeography of this ancient landmass.

## Supplemental Information

10.7717/peerj.6457/supp-1 Supplemental Information 1 Table S1. Details for 3D scans of specimens.

10.7717/peerj.6457/supp-2 Supplemental Information 2 Raw sequence data.

## Additional Information and Declarations

### Field Study Permissions

The following information was supplied relating to field study approvals (i.e., approving body and any reference numbers): The state forest departments of Kerala provided permission for fieldwork (WL12-6574/2006).

### DNA Deposition

The following information was supplied regarding the deposition of DNA sequences: Our new sequences are available in GenBank: MK144662, MK153264–MK153268. The raw sequence data are available as a Supplemental File (FASTA).

### Data Availability

The following information was supplied regarding data availability: All data, code, and results are available at the Dryad repository: DOI 10.5061/dryad.4q3d25m. All image stacks, associated metadata, and surface models utilized in this study are available online via MorphoSource (Table S1).

### New Species Registration

The following information was supplied regarding the registration of a newly described species: Publication LSID: urn:lsid:zoobank.org:pub:5ADE399C-37CC-4821-BD81-15F420731BB7. *Astrobatrachinae* Vijayakumar, Pyron, Dinesh, Torsekar, Srikanthan, Swamy, Stanley, Blackburn & Shanker, In Press. LSID: urn:lsid:zoobank.org:act:B43D4B4E-FC28-4E76-B93A-FB4B81975933. *Astrobatrachus* Vijayakumar, Pyron, Dinesh, Torsekar, Srikanthan, Swamy, Stanley, Blackburn & Shanker, In Press. LSID: urn:lsid:zoobank.org:act:28F98D95-3E1E-41BB-B7A1-CC6766432E22. *Astrobatrachus kurichiyana* Vijayakumar, Pyron, Dinesh, Torsekar, Srikanthan, Swamy, Stanley, Blackburn & Shanker, In Press. LSID: urn:lsid:zoobank.org:act:A4A21F51-2A11-41C6-AA2A-B6F390070868.

## References

[ref-1] Abraham RK, Pyron RA, Ansil BR, Zachariah A, Zachariah A (2013). Two novel genera and one new species of treefrog (Anura: Rhacophoridae) highlight cryptic diversity in the Western Ghats of India. Zootaxa.

[ref-2] Anderson J (1871). A list of the reptilian accession to the Indian Museum, Calcutta from 1865 to 1870, with a description of some new species. Journal of the Asiatic Society of Bengal.

[ref-3] Batsch AJGK (1796). Umriß der gesammten Naturgeschichte: ein Auszug aus den frühern Handbüchern des Verfassers für seine Vorfesungen.

[ref-4] Beaupre SJ, Jacobsen ER, Lillywhite HB, Zamudio K (2004). Guidelines for use of live amphibians and reptiles in field and laboratory research, 2nd edition. Revised by the Herpetological Animal Care and Use Committee (HACC) of the American Society of Ichthyologists and Herpetologists. www.asih.org/files/hacc-final.pdf.

[ref-5] Beddome RH (1878). Description of a new batrachian from southern India, belonging to the family Phryniscidae. Proceedings of the Zoological Society of London.

[ref-6] Biju SD, Bossuyt F (2003). New frog family from India reveals an ancient biogeographical link with the Seychelles. Nature.

[ref-66] Biju SD, Bocxlaer IV, Varad G, Roelants K, Nagaraju J, Bossuyt F (2007). A new nightfrog, Nyctibatrachus minimus sp. nov. (Anura: Nyctibatrachidae): the smallest frog from India. Current Science.

[ref-7] Biju SD, Garg S, Mahony S, Wijayathilaka N, Senevirathne G, Meegaskumbura M (2014). DNA barcoding, phylogeny and systematics of Golden-backed frogs (*Hylarana*, Ranidae) of the Western Ghats-Sri Lanka biodiversity hotspot, with the description of seven new species. Contributions to Zoology.

[ref-8] Biju SD, Roelants K, Bossuyt F (2008). Phylogenetic position of the montane treefrog *Polypedates variabilis* Jerdon, 1853 (Anura: Rhacophoridae), and description of a related species. Organisms Diversity & Evolution.

[ref-9] Biju SD, Van Bocxlaer I, Giri VB, Loader SP, Bossuyt F (2009). Two new endemic genera and a new species of toad (Anura: Bufonidae) from the Western Ghats of India. BMC Research Notes.

[ref-10] Biju SD, Van Bocxlaer I, Mahony S, Dinesh KP, Radhakrishnan C, Zachariah A, Giri VB (2011). A taxonomic review of the Night frog genus *Nyctibatrachus* Boulenger,1882 in the Western Ghats, India (Anura: Nyctibatrachidae) with description of twelve new species. Zootaxa.

[ref-11] Blommers-Schlösser RMA (1993). Systematic relationships of the Mantellinae Laurent 1946 (Anura Ranoidea). Ethology Ecology & Evolution.

[ref-12] Bossuyt F, Brown RM, Hillis DM, Cannatella DC, Milinkovitch MC (2006). Phylogeny and biogeography of a cosmopolitan frog radiation: Late Cretaceous diversification resulted in continent-scale endemism in the family Ranidae. Systematic Biology.

[ref-13] Bossuyt F, Meegaskumbura M, Beenaerts N, Gower DJ, Pethiyagoda R, Roelants K, Mannaert A, Wilkinson M, Bahir MM, Manamendra-Arachchi K, Ng PKL, Schneider CJ, Oommen OV, Milinkovitch MC (2004). Local endemism within the Western Ghats-Sri Lanka biodiversity hotspot. Science.

[ref-14] Bossuyt F, Milinkovitch MC (2000). Convergent adaptive radiations in Madagascan and Asian ranid frogs reveal covariation between larval and adult traits. Proceedings of the National Academy of Sciences of the United States of America.

[ref-15] Bossuyt F, Milinkovitch MC (2001). Amphibians as indicators of early tertiary “out-of-India” dispersal of vertebrates. Science.

[ref-16] Boulenger GA (1882). Catalogue of the Batrachia Salientia s. Ecaudata in the collection of the British museum.

[ref-17] Boulenger GA (1888). Note on the classification of the Ranidae. Proceedings of the Zoological Society of London.

[ref-18] Chandramouli SR, Dutta SK (2015). Comparative osteology of anuran genera in the Western Ghats, Peninsular India. Alytes.

[ref-19] Dahanukar N, Modak N, Krutha K, Nameer PO, Padhye AD, Molur S (2016). Leaping frogs (Anura: Ranixalidae) of the Western Ghats of India: an integrated taxonomic review. Journal of Threatened Taxa.

[ref-20] Delorme M, Dubois A, Kosuch J, Vences M (2004). Molecular phylogenetic relationships of *Lankanectes corrugatus* from Sri Lanka: endemism of South Asian frogs and the concept of monophyly in phylogenetic studies. Alytes.

[ref-64] De Queiroz K (2007). Species concepts and species delimitation. Systematic Bology.

[ref-21] Dinesh KP, Vijayakumar SP, Channakesavamurthy BH, Torsekar VR, Kulkarni NU, Shanker K (2015). Systematic status of *Fejervarya* (Amphibia, Anura, Dicroglossidae) from South and SE Asia with the description of a new species from the Western Ghats of Peninsular India. Zootaxa.

[ref-22] Dubois A (1986). Diagnose préliminaire d“un nouveau genre de Ranoidea (Amphibiens, Anoures) du sud de l”Inde. Alytes.

[ref-23] Dubois A (1987). Miscellanea taxinomica batrachologica (I). Alytes.

[ref-24] Dubois A (2005). Amphibia Mundi. 1.1. An ergotaxonomy of recent amphibians. Alytes.

[ref-25] Dubois A, Ohler A (2001). A new genus for an aquatic ranid (Amphibia, Anura) from Sri Lanka. Alytes.

[ref-26] Dubois A, Ohler A, Biju SD (2001). A new genus and species of Ranidae (Amphibia, Anura) from south-western India. Alytes.

[ref-27] Dutta SK, Vasudevan K, Chaitra M, Shanker K, Aggarwal RK (2004). Jurassic frogs and the evolution of amphibian endemism in the Western Ghats. Current Science.

[ref-28] Feng Y-J, Blackburn DC, Liang D, Hillis DM, Wake DB, Cannatella DC, Zhang P (2017). Phylogenomics reveals rapid, simultaneous diversification of three major clades of Gondwanan frogs at the Cretaceous–Paleogene boundary. Proceedings of the National Academy of Sciences of the United States of America.

[ref-29] Fjeldsaå J, Lovett JC (1997). Geographical patterns of old and young species in African forest biota: the significance of montane areas as evolutionary centres. Biodiversity and Conservation.

[ref-30] Frost DR, Grant T, Faivovich J, Bain RH, Haas A, Haddad CFB, De Sá RO, Channing A, Wilkinson M, Donnellan SC, Raxworthy CJ, Campbell JA, Blotto BL, Moler P, Drewes RC, Nussbaum RA, Lynch JD, Green DM, Wheeler WC (2006). The amphibian tree of life. Bulletin of the American Museum of Natural History.

[ref-31] Garg S, Suyesh R, Sukesan S, Biju SD (2017). Seven new species of Night Frogs (Anura, Nyctibatrachidae) from the Western Ghats Biodiversity Hotspot of India, with remarkably high diversity of diminutive forms. PeerJ.

[ref-32] Gray JE (1825). A synopsis of the genera of reptiles and Amphibia, with a description of some new species. Annals of Philosophy.

[ref-33] Günther A (1858). On the systematic arrangement of the tailless batrachians and the structure of *Rhinophrynus dorsalis*. Proceedings of the Zoological Society of London.

[ref-34] Günther ACLG (1876). Third report on collections of Indian reptiles obtained by the British Museum. Proceedings of the Zoological Society of London.

[ref-35] Hoffman AC (1932). Researches relating to the validity of the South African Polypedatidae (Rhacophoridae) as an autonomous family of the Anura. South African Journal of Science.

[ref-36] Jetz W, Pyron RA (2018). The interplay of past diversification and evolutionary isolation with present imperilment across the amphibian tree of life. Nature Ecology & Evolution.

[ref-37] Katoh K, Misawa K, Kuma KI, Miyata T (2002). MAFFT: a novel method for rapid multiple sequence alignment based on fast Fourier transform. Nucleic Acids Research.

[ref-38] Kozlov AM, Aberer AJ, Stamatakis A (2015). ExaML version 3: a tool for phylogenomic analyses on supercomputers. Bioinformatics.

[ref-62] Lanfear R, Calcott B, Kainer D, Mayer C, Stamatakis A (2014). Selecting optimal partitioning schemes for phylogenomic datasets. BMC evolutionary biology.

[ref-39] Lanfear R, Frandsen PB, Wright AM, Senfeld T, Calcott B (2017). PartitionFinder 2: new methods for selecting partitioned models of evolution for molecular and morphological phylogenetic analyses. Molecular Biology and Evolution.

[ref-40] Laurent RF, Grassé P, Delsol M (1986). Sous Classe des Lissamphibiens (Lissamphibia). Traité de Zoologie. Anatomie, Systematique, Biologie.

[ref-41] Mani M (1974). Ecology and biogeography in India.

[ref-42] Meegaskumbura M, Bossuyt F, Pethiyagoda R, Manamendra-Arachchi K, Bahir M, Milinkovitch MC, Schneider CJ (2002). Sri Lanka: an amphibian hot spot. Science.

[ref-65] Peters WCH (1863). Über eine neue Schlangen-Gattung, Styporhynchus, und verschiedene andere Amphibien des zoologischen Museum. Monatsberichte der Königlichen Preussische Akademie des Wissenschaften zu Berlin.

[ref-43] Prasad V, Farooqui A, Tripathi SKM, Garg R, Thakur B (2009). Evidence of Late Palaeocene-Early Eocene equatorial rain forest refugia in southern Western Ghats, India. Journal of Biosciences.

[ref-44] Pyron RA (2014). Biogeographic analysis reveals ancient continental vicariance and recent oceanic dispersal in amphibians. Systematic Biology.

[ref-45] Pyron RA, Burbrink FT, Colli GR, de Oca ANM, Vitt LJ, Kuczynski CA, Wiens JJ (2011). The phylogeny of advanced snakes (Colubroidea), with discovery of a new subfamily and comparison of support methods for likelihood trees. Molecular Phylogenetics and Evolution.

[ref-46] Pyron RA, Wiens JJ (2011). A large-scale phylogeny of Amphibia including over 2800 species, and a revised classification of extant frogs, salamanders, and caecilians. Molecular Phylogenetics and Evolution.

[ref-47] Roelants K, Gower DJ, Wilkinson M, Loader SP, Biju SD, Guillaume K, Moriau L, Bossuyt F (2007). Global patterns of diversification in the history of modern amphibians. Proceedings of the National Academy of Sciences of the United States of America.

[ref-48] Roelants K, Jiang J, Bossuyt F (2004). Endemic ranid (Amphibia: Anura) genera in southern mountain ranges of the Indian subcontinent represent ancient frog lineages: evidence from molecular data. Molecular Phylogenetics and Evolution.

[ref-49] Senevirathne G, Samarawickrama VAMPK, Wijayathilaka N, Manamendra-Arachchi K, Bowatte G, Samarawickrama DRNS, Meegaskumbura M (2018). A new frog species from rapidly dwindling cloud forest streams of Sri Lanka—*Lankanectes pera* (Anura, Nyctibatrachidae). Zootaxa.

[ref-50] Shanker K, Vijayakumar SP, Ganeshaiah KN (2017). Unpacking the species conundrum: philosophy, practice and a way forward. Journal of Genetics.

[ref-51] Stamatakis A (2006). RAxML-VI-HPC: maximum likelihood-based phylogenetic analyses with thousands of taxa and mixed models. Bioinformatics.

[ref-52] Stamatakis A (2014). RAxML version 8: a tool for phylogenetic analysis and post-analysis of large phylogenies. Bioinformatics.

[ref-63] Trueb L, Diaz R, Blackburn DC (2011). Osteology and chondrocranial morphology of Gastrophryne carolinensis (Anura: Microhylidae), with a review of the osteological diversity of New World microhylids. Phyllomedusa.

[ref-53] Van Bocxlaer I, Biju SD, Loader SP, Bossuyt F (2009). Toad radiation reveals into-India dispersal as a source of endemism in the Western Ghats-Sri Lanka biodiversity hotspot. BMC Evolutionary Biology.

[ref-54] Van Bocxlaer I, Biju SD, Willaert B, Giri VB, Shouche YS, Bossuyt F (2012). Mountain-associated clade endemism in an ancient frog family (Nyctibatrachidae) on the Indian subcontinent. Molecular Phylogenetics and Evolution.

[ref-55] van der Meijden A, Vences M, Meyer A (2004). Novel phylogenetic relationships of the enigmatic brevicipitine and scaphiophrynine toads as revealed by sequences from the nuclear *Rag–1* gene. Proceedings of the Royal Society of London. Series B: Biological Sciences.

[ref-56] Vences M, Wanke S, Odierna G, Kosuch J, Veith M (2000). Molecular and karyological data on the south Asian ranid genera *Indirana*, *Nyctibatrachus* and *Nannophrys* (Anura: Ranidae). Hamadryad.

[ref-57] Vijayakumar SP, Dinesh KP, Prabhu MV, Shanker K (2014). Lineage delimitation and description of nine new species of bush frogs (Anura: *Raorchestes*, Rhacophoridae) from the Western Ghats Escarpment. Zootaxa.

[ref-58] Vijayakumar SP, Menezes RC, Jayarajan A, Shanker K (2016). Glaciations, gradients, and geography: multiple drivers of diversification of bush frogs in the Western Ghats Escarpment. Proceedings of the Royal Society B: Biological Sciences.

[ref-59] Wallace AR (1876). The geographical distribution of animals with a study of the relations of living and extinct faunas as elucidating the past changes of the earth’s surface.

[ref-60] Wiens JJ, Sukumaran J, Pyron RA, Brown RM (2009). Evolutionary and biogeographic origins of high tropical diversity in Old World frogs (Ranidae). Evolution.

[ref-61] Yuan Z-Y, Zhang B-L, Raxworthy CJ, Weisrock DW, Hime PM, Jin J-Q, Lemmon EM, Lemmon AR, Holland SD, Kortyna ML, Zhou W-W, Peng M-S, Che J, Prendini E (2018). Natatanuran frogs used the Indian Plate to step-stone disperse and radiate across the Indian Ocean. National Science Review.

